# Functional Food and Bioactive Compounds on the Modulation of the Functionality of HDL-C: A Narrative Review

**DOI:** 10.3390/nu13041165

**Published:** 2021-04-01

**Authors:** Karla Paulina Luna-Castillo, Sophia Lin, José Francisco Muñoz-Valle, Barbara Vizmanos, Andres López-Quintero, Fabiola Márquez-Sandoval

**Affiliations:** 1 Doctorado en Ciencias de la Nutrición Traslacional, Departamento de Clínicas de la Reproducción Humana, Crecimiento y Desarrollo Infantil, Centro Universitario de Ciencias de la Salud, Universidad de Guadalajara, Guadalajara 44340, Mexico; karla.luna3337@alumnos.udg.mx (K.P.L.-C.); drjosefranciscomv@cucs.udg.mx (J.F.M.-V.); bvizmanos@yahoo.com.mx (B.V.); 2 School of Population Health, University of New South Wales, Sydney, NSW 2052, Australia; sophia.lin@unsw.edu.au; 3 Instituto de Investigación en Ciencias Biomédicas (IICB), Centro Universitario de Ciencias de la Salud, Universidad de Guadalajara, Guadalajara 44340, Mexico; 4 Instituto de Nutrigenética y Nutrigenómica Traslacional, Centro Universitario de Ciencias de la Salud, Universidad de Guadalajara, Guadalajara 44340, Mexico

**Keywords:** HDL, dietary compounds, polyphenols, functional food, bioactive compounds, cardiovascular disease, high-density lipoprotein functionality, HDL functionality

## Abstract

Cardiovascular diseases (CVD) remain a serious public health problem and are the primary cause of death worldwide. High-density lipoprotein cholesterol (HDL-C) has been identified as one of the most important molecules in the prevention of CVD due to its multiple anti-inflammatories, anti-atherogenic, and antioxidant properties. Currently, it has been observed that maintaining healthy levels of HDL-C does not seem to be sufficient if the functionality of this particle is not adequate. Modifications in the structure and composition of HDL-C lead to a pro-inflammatory, pro-oxidant, and dysfunctional version of the molecule. Various assays have evaluated some HDL-C functions on risk populations, but they were not the main objective in some of these. Functional foods and dietary compounds such as extra virgin olive oil, nuts, whole grains, legumes, fresh fish, quercetin, curcumin, ginger, resveratrol, and other polyphenols could increase HDL functionality by improving the cholesterol efflux capacity (CEC), paraoxonase 1 (PON1), and cholesteryl ester transfer protein (CETP) activity. Nevertheless, additional rigorous research basic and applied is required in order to better understand the association between diet and HDL functionality. This will enable the development of nutritional precision management guidelines for healthy HDL to reduce cardiovascular risk in adults. The aim of the study was to increase the understanding of dietary compounds (functional foods and bioactive components) on the functionality of HDL.

## 1. Introduction

Cardiovascular diseases (CVD) remain a serious public health problem and are a primary cause of death worldwide [1]. Global mortality trends from CVD have increased over the last 10 years by 6.4% from 225.41 to 239.9 per 100,000 between 2009 and 2019 [2]. Several studies have indicated that healthy HDL-C levels (40–60 mg/dL in men, 50–60 mg/dL in women) [3] are associated with a lower risk of incident CVD [4,5,6], while low levels of HDL-C (<35 mg/dL in each sex) have been associated with an increased risk [3,5,7]. According to the National Health and Nutrition Examination Survey 2015–2018 in the United States, the prevalence of low HDL-C among adults over 19 aged was 17.2% in 2015–2018 and was higher among aged 20–29 by 17.6% and 40–59 aged by 18.5% and 60 and over by 14.6% [8]. Similarly, European [9], Asia [10], and Africa [11] populations have a prevalence of 22.1% (50 years or older), 20.4% (18–69 years), and 28.6% (28–73 years), respectively. Further, in Latin America, the reported prevalence of low HDL-C is higher by 27.5% in young people, higher among aged 29 and over by 29.5% [12]. This type of dyslipidemia is the commonest form worldwide. However, maintaining healthy HDL-C levels does not seem to be sufficient in lowering CVD risk if the functionality of this particle is not adequate, especially its cholesterol efflux capacity (CEC). This lipoprotein is essential in several cardioprotective mechanisms, including reverse cholesterol transport (RCT), CEC, stimulation of endothelial nitric oxide (NO) (vasodilatory capacity), inhibition of reactive oxygen species (ROS) and decreasing low-density lipoprotein cholesterol (LDL-C) oxidation (antioxidant capacity) and inhibiting the expression of adhesion molecules on endothelial cells [13,14,15,16].

Modifications in the structure and composition of HDL-C in the body can result in dysfunctional particles, losing its atheroprotective properties and developing pro-inflammatory and pro-oxidant characteristics [14,17,18]. Risk factors for abnormal HDL-C include low-grade inflammation [13,14,19], acute phase response [20], diabetes [21], obesity, ethnicity [22], and poor-quality diet [23,24]. Exercise is related to the improvement in HDL concentrations and functionality [25,26], while diet can have both positive and negative effects on HDL-C functionality [24,27,28]. Studies have shown that a healthy diet, characterized by high consumption of fruits, vegetables, legumes, fish, nuts, and olive oil, could increase the number of HDL-C particles [27,29,30]. Dietary functional food in the Mediterranean diet (MD), such as olive oil, whole grains, nuts, legumes, and fish, could contribute to increased CEC, HDL-C esterification index, and paraoxonase 1 (PON1) activity, according to a subsample from the PREDIMED Study (PREvención con DIeta MEDiterránea) [27,29].

In the last 10 years, there has been increasing evidence for the benefits of functional foods and bioactive compounds on CEC, PON1 activation and expression, and cholesteryl ester transfer protein (CETP) activity. Nonetheless, there is currently insufficient evidence to inform the development of a precision nutritional guideline for the treatment and prevention of HDL concentration and functionality. According to the current American College of Cardiology/American Heart Association (ACC/AHA) [31] and European Society of Cardiology (ESC) and European Atherosclerosis Society (EAS) Guidelines for the management of dyslipidemias, no specific goals for HDL-C levels have been determined in clinical trials, although increases in HDL-C predicts atherosclerosis, and low HDL-C is associated with incident and mortality in coronary artery disease, even at low LDL concentrations [32,33].

Experts in the areas of nutrition and metabolism, genetics, as well as immunology are represented in this narrative review. It serves as an integrative approach to figure out the importance of HDL-C in the pathophysiology of CVD, its atheroprotective properties, and the current research about the dietary compounds which contribute to increased HDL-C functionality. Therefore, we overviewed the HDL-C principal cardioprotective properties in halting the progression of atherosclerosis and the structural and functional changes to which the HDL particle may be exposed during inflammation. Moreover, the main aim was to present the available evidence to increase the understanding of dietary compounds (functional foods and bioactive components) on the functionality of HDL (CEC, PON1 activation and expression, CETP activity, and expression of adhesion molecules).

## 2. Materials and Methods

The methodological approach for the present narrative review was performed using the PubMed^®^ and Web of ScienceTM databases updated to 24 March 2021. The search was carried out from February 2020 to March 2021. Search terms included in the title and abstract were “(antioxidants or functional foods or bioactive compounds) AND HDL functionality”, “(antioxidants or functional foods or bioactive compounds) AND cholesterol efflux“, “(antioxidant or functional foods for bioactive compounds) AND (PON1 or paraoxonase 1) AND HDL”, “(antioxidants or functional foods or bioactive compounds) AND (CETP or cholesteryl ester transfer protein)” and “(Antioxidants or functional foods or bioactive compounds) AND endothelial markers and HDL”. For specific food and dietary compounds (identified in the first scrutiny) the following terms were also applied after the dietary compound name AND: “HDL functionality”; “cholesterol efflux capacity”; “CETP OR cholesterol ester transfer protein”; “PON1 OR paraoxonase 1”; “adhesion molecules”; “VCAM”; “ICAM”; “E-selectin”. This narrative review represents a summary of evidence of the last 10 years (2011–2021). Hand-searching of the reference list of each article was conducted to ensure that no important research articles were missed. We highlight the included clinical trials were screened to the more recent publications, in the last 5 years, in order to show the latest evidence oriented to translational knowledge in humans.

We selected all studies in English full text, available in human studies, in vivo and in vitro studies, with main results about one or more of the four HDL-C functions: (1) the inhibition of the oxidation of oxidized LDL (ox-LDL) through the activity of PON1, (2) the CETP activity, (3) the CEC, and (4) the inhibition of the expression of adhesion molecules (VCAM-1, ICAM-1, E-selectin). The methodology and eligibility of all articles were analyzed carefully by the authors. The lack of consistency to relate the dietary compounds and HDL-C functionality was considered an exclusion criterion.

This review was not conducted systematically because there was not enough consistent evidence about this topic.

## 3. Pathophysiology of Cardiovascular Disease and HDL

The leading cause of CVD incidence and mortality is atherosclerosis [34], a chronic inflammatory disease characterized by the hardening of the arterial walls, due to the progressive accumulation of lipid plaques and inflammatory cells within the intima of the large and mid-sized arteries, resulting in decreased or absent blood flow [35,36,37].

**Figure 1:**
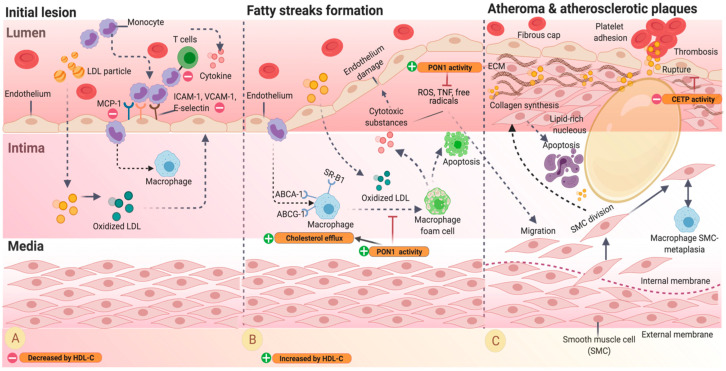
Progression of atherosclerosis adapted from [19,40,41,42]. (**A**) The excess of LDL-C particles in the vascular endothelium results in the internalization of lipids in the intima, which are oxidized, stimulating local activating leukocyte migration to the intima. Oxidized LDL-C triggers adhesion molecules, including ICAM-1, VCAM-1, E-selectin, and MCP-1 in the endothelial cells during the first steps of the lesion. HDL-C inhibits the expression of adhesion molecules (VCAM-1, ICAM-1, E-selectin) (orange rounded rectangle and green plus sign). (**B**) After penetrating the arterial wall, monocytes uptake ox-LDL, and form a macrophage foam cell, contributing to the formation of lipid streaks. HDL-C inhibits the oxidation of ox-LDL through the activity of PON1. (**C**) Cytotoxic substances stimulate smooth muscle cells (SMC) migration into the intima where it is divided to synthesize collagen, and the extracellular matrix (ECM) creates a fibrous cap and stabilizes the plaque. HDL-C decreases CETP activity, which improves the stability of the plaque and to avoid the formation of thrombus. Abbreviations: LDL-particle, low-density lipoprotein particle; ICAM-1, intercellular adhesion molecule 1; VCAM-1, vascular adhesion molecule 1; MCP-1, macrophage chemoattractant protein 1; ABCA-1, ATP-binding cassette transporter A member 1; ABCG-1, ATP-binding cassette transporter G member 1; SR-B1, scavenger receptor class B type 1; ROS, reactive oxygen species; TNF, tumor necrosis factor; SMC, smooth muscle cells.

The atherosclerosis processes include the formation of fatty streaks, atheroma, and atherosclerotic plaques (Figure 1) [38].

The first observable event in the process of atherosclerosis is the accumulation of plaque (lipids, calcium, and fibrin) [39]. Hypercholesterolemia results in the internalization of lipids, especially LDL-C in the intima, creating changes in the permeability of the arterial endothelium with concomitant endothelial dysfunction [35,36]. The vascular endothelium is a semipermeable barrier that, under normal conditions, controls the diffusion of plasma molecules and regulates vascular tone, inflammation, and prevents thrombus formation. A dysfunctional endothelium results in the loss of these functions [35]. These factors lead to cell oxidation of the trapped particles [38]. Ox-LDL stimulates the local secretion of cytokines, activating leukocyte migration to the intima. Thus, circulating monocytes and T-cells are recruited to attach to the vascular endothelium and adherence to endothelial cells that express adhesion molecules. These include intercellular adhesion molecule-1 (ICAM-1), vascular adhesion molecule-1 (VCAM-1), selectins, and chemotactic signals like monocyte chemoattractant protein 1 (MCP-1), IL-8, and gamma interferon-inducible protein 10. The endothelial cells then infiltrate into the intima (Figure 1A) [35,36]. In the initial injury, HDL-C participates with the inhibition of the expression of adhesion molecules: VCAM-1, ICAM-1 and E-selectin (encoded by *VCAM1*, *ICAM1* and *SELE* genes, respectively). (Figure 1B, orange rounded rectangle and red minus sign).

After penetrating the arterial wall in response to chemotaxis, monocytes are stimulated by the macrophage colony-stimulating factor (M-CSF) that increases their expression of collecting receptors. M-CSF uptake ox-LDL during the monocyte-to-macrophage differentiation to foam cells, promoting plaque progression (Figure 1B) [35,38]. Accumulation of foam cells on the arterial walls contributes to the formation of lipid streaks in early steps [35,38]. Some foam cells undergo apoptosis during the intima lesion, producing a lipid-rich necrotizing nucleus in the atherosclerotic plaque. Macrophage foam cells can also produce cytotoxic substances such as tumor necrosis factor (TNF), growth factor, pre-coagulation substances (tissue factors), reactive oxygen species (ROS), such as superoxide anion (O_2−_), and free radicals, which lead to more endothelial damage [38]. These substances further stimulate vascular smooth muscle cell migration from the media into the intima, where they divide and produce extracellular matrix components such as collagen and fibrous caps [35]. Particles of HDL-C in this phase can increase CEC and the inhibition of the oxidation of ox-LDL through the activity of PON1 (Figure 1B, orange rounded rectangle and green plus sign).

In later stages, calcification can occur, and fibrosis could appear (Figure 1C) [38]. The progression of atherosclerotic plaques is also characterized by a decrease in vascular smooth muscle cells and the formation of immature and leaking vessels, increasing its susceptibility to rupture. The alterations on the plaque initiate platelet adhesion and activation of the coagulation cascade, allowing the formation of thrombus and clinical manifestations of atherosclerotic diseases such as acute myocardial infarction or sudden death [35,40]. HDL-C has also an important role in this part: it encourages a decrease in the CETP activity (Figure 1C, orange rounded rectangle and red minus sign).

## 4. HDL-C Physiology and Pathophysiology

Current evidence suggests that maintaining healthy HDL-C levels is insufficient when circulating HDL-C is dysfunctional [15,43]. The main cardioprotective properties of HDL are the CEC [6,13,44], the inhibition of the oxidation of ox-LDL through the activity of PON1, the inhibition of the expression of adhesion molecules (VCAM-1, ICAM-1, E-selectin), and the CETP activity [24,45,46].

HDL-C particles and apolipoprotein A-I (apoA-I) confer protection against atherosclerosis through different pathways and mechanisms [15,43]. The most recognized and studied cardioprotective property associated with HDL-C molecules is their key function in RCT [6,44], that is, HDL-C particles capacity to transport cholesterol from extrahepatic tissues to the liver for processing or excretion from the body [6,47,48]. RCT includes two main steps; the first one, lipid-free, or lipid-poor apoA-I (pre-β HDL), binds to ATP-binding cassette transporter A member 1 (ABCA-1) in macrophages and accepts cholesterol from membranes of lipid-loaded cells of the arterial wall [44,49]. The release of free cholesterol on HDL surfaces is esterified by lecithin cholesterol acyltransferase (LCAT) and generates a mature HDL particle (α-HDL). RCT is completed at the last step when esterified cholesterol is delivered to the liver by the binding scavenger receptor class B type 1 (SR-B1) [44].

In particular, the macrophage cholesterol efflux (CE), a small part of the RCT [13], is characterized by the ability of HDL-C to extract excessive cholesterol from peripheral membranes from macrophages and foam cells. This function is carried out by different mechanisms involving transporters such as ATP-binding cassette transporter A member 1 (ABCA-1), ATP-binding cassette subfamily G (ABCG-1), and ATP-binding cassette subfamily G member 4 (ABCG-4) [50,51]. The macrophage CE is mediated most effectively by cholesterol-deficient and phospholipid-deficient apoA-I complexes and by very small HDL particles [15].

There are other potentially favorable properties of HDL-C including anti-inflammatory, antithrombotic, and antioxidant effects [24,45,46]. HDL-C can also reduce endothelial dysfunction [15] with additional antiatherogenic and antioxidant properties by inhibiting LDL-C oxidation and stimulating the synthesis of nitric oxide (NO), a powerful vasodilator, and inhibiting reactive ROS [15,17]. Therefore, HDL can decrease the thrombotic risk associated with increased synthesis and bioavailability of endothelial NO and prostacyclin, preventing platelet activation, decreasing thrombin generation, and consequently downregulating platelet aggregation, and as a precursor of fibrin synthesis [52]. Another anti-inflammatory action of HDL-C is the hydrolysis of lipids oxidized by enzymes associated with HDL-C, such as platelet-activating factor-acetylhydrolase (PAF-AH), PON-1, apoA-I, LCAT, and CETP [46,53]. HDL-C has been shown to protect against LDL-C oxidation by preventing the generation of pro-inflammatory lipids such as lipid hydroperoxides and oxidized phospholipids [54]. In particular, PON1 appears to be responsible for decreasing the intensity of oxidative damage to macrophages, stimulating the cholesterol flow, and attenuating the oxidative stress in atherosclerotic lesions [55].

Furthermore, HDL reduces coronary atherosclerosis by decreasing expression of *VCAM1, ICAM1* and *SELE* genes in endothelial cells, key mediators in the passage of immunocompetent cells from the blood capillaries to the inflammation site in the arterial wall, thereby reducing inflammation and inhibiting the oxidation of LDL-C [17,56]. There is additional evidence that HDL-C associated with proteins such as apoAII, apoAIV, apoJ, and apoF can inhibit the expression and circulating levels of VCAM-1, ICAM-1, and E-selectin [57]. HDL-C modulates the chronic inflammatory response, inhibiting IL-1β and TNFα production by blocking the interaction of monocytes with activated T cells, the primary mechanism of monocyte stimulation [17,58,59]. Contact between activated T cells and monocytes or macrophages drives the production of several cytokines (IL-1β, IL-6, IL-8, MCP1, and TNFα), responsible for atherogenesis and tissue destruction [17,59]. In the same way, HDL-C may attenuate the dysregulated apoptosis of atherosclerotic plaque cells by inhibiting macrophage and endothelial cell apoptosis by different stimulations [44].

**Figure 2:**
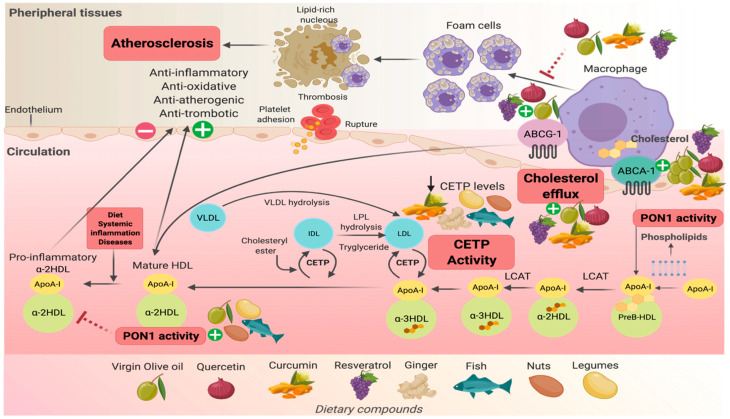
Molecular mechanism of functional foods that enhance HDL functionality adapted from [65,66]. Virgin olive oil, quercetin, and resveratrol participate and contribute to the improvement of cholesterol efflux from macrophages by increasing the expression of *ABCG1* and *ABCA1* transporters, while curcumin only influences the *ABCA1* transporter. All of these mechanisms would prevent the accumulation of cholesterol within macrophages in the arterial wall, and its subsequent relationship with the progression of atherosclerosis. Quercetin is related to the increase in PON1 protein and its expression, improving the anti-oxidant, anti-inflammatory, and anti-atherogenic activity of HDL. Abbreviations: ABCG-1, ATP-binding cassette sub-family G member 1; ABCA-1, ATP binding cassette subfamily A member 1; PON1, paraoxonase 1; CETP, cholesteryl ester transfer protein; LCAT, lecithin-cholesterol acyltransferase; HDL, high-density lipoprotein; LDL, low-density lipoprotein; VLDL, very-low-density lipoprotein; IDL, intermediate-density lipoprotein.

Other cardioprotective capacities of HDL-C have been related to HDL-lipoprotein-associated phospholipase A2 (HDL-LpPLA2). The activity of HDL-LpPLA2 has been associated with lower cardiovascular risk in clinical studies [60]. Moreover, LpPLA2 when bound to LDL exerts pro-inflammatory and atherogenic effects [61]. The complexity of HDL-C remodeling regarding the structure, size, shape, and density characteristics has been previously described [62]. There exist multiple classifications according to the employed techniques. Respecting shape, alpha subfractions (alphaHDL) are actively involved in the RCT, and they represent the most abundant circulating HDL-C particles (Figure 2). HDL subfractions confer different properties that are inherent to their composition [63,64].

### 4.1. Factors That Impact the Function of HDL

Functionality and composition of HDL-C particles can be affected under certain circumstances [13] such as the acute phase response after infection, chronic low-grade inflammation, acute myocardial infarctions, atherogenesis [20], diabetes mellitus, obesity, and metabolic syndrome (MS) [21,22], ethnicity [22], smoking [15], and diet [23,67]. All these factors can produce an atherogenic and pro-inflammatory HDL-C, in other words, HDL-C can become dysfunctional [15,19,24].

Dysfunctional lipoprotein results from the alteration of the proteins, enzymes, or transporters involved in HDL metabolism, which affects the process of maturation and remodeling of HDL [44]. Modifications in arginine, lysine, and methylglyoxal, covalently modified proteins by non-enzymatic glycation reactions, have been related to the loss of apoA-I ability to activate LCAT. The latter results in the inability of apoA-I to inhibit the infiltration of neutrophils into the intima of the carotid arteries in animal models. Exposure of the recombinant HDL-C to methylglyoxal resulting in the loss of anti-inflammatory activities, as occurs in apoA-I, produces dysfunctional HDL-C [17,68,69].

Similarly, myeloperoxidase (MPO), a heme protein overexpressed in human atherosclerosis lesions, can damage HDL-C particles. High levels of MPO have been detected during the progression of atherosclerosis [44]. Lipid-free apoA-I is oxidized by MPO in vitro, and the macrophage CE by ABCA-1 pathway is decreased. Oxidation of methionine in apoA-I associated with HDL-C also disturbs the capacity to activate LCAT [43].

### 4.2. Effect of Inflammation on HDL

During inflammation, HDL-C has been found to undergo significant changes in size, composition, and structure. Some enzymes participate in the remodeling of HDL-C due to inflammation, specifically serum amyloid A (SAA) and secretory phospholipase A2 (sPLA2-IIA) [15,18].

The reduction in HDL-C concentration during the acute response has been linked to two main mechanisms: the decrease in the liver synthesis of apoA-I [17] and the replacement of apoA-I with SAA in HDL-C particles, due to its overexpression through transactivation of nuclear activation factor-kappa β (NFk- β). This last mechanism modifies the expression of *apoA-I* and *PON1*, inhibits the activation of peroxisome proliferation-activated receptors (PPARs), which convert the HDL-C into an inflammatory particle, reducing its ability to inhibit LDL oxidation [14,15,18]. Additionally, lipopolysaccharides (LPS) and inflammatory cytokines inhibit cholesterol output from cells by reducing the expression of the *ABCA-1* gene and increasing the concentration of intracellular cholesterol. Interleukin 1 (IL-1) also reduces the expression of *PPAR* and liver X receptor (*LXR*) [70].

SAA is associated with an inflammatory response mechanism, from the stimulation the production of inflammatory factor as monocytes and macrophages and immune cells migrations as MCP-1 in human monocytes, causing the vascular inflammation by expression of oxidized LDL receptor 1 [17]. Together, this process contributes to the development of atherosclerotic plaque.

Inflammation promotes a reduction in LCAT activity. During the acute phase response and inflammation, there is a reduction in mRNA expression and *CETP* and phospholipid-transfer protein (PLTP) activity, contributing to changes in HDL-C metabolism and function [70,71]. In the same way, there is a reduction in the antioxidant capacity due to a decrease in other proteins associated with HDL: PON1 and PAF-AH [44].

All changes in RCT enzymes lead to lower HDL-C levels that have faster catabolism and are removed from circulation [70]. This produces deterioration in one of the main functions of HDL-C, the RCT capacity [44], a function considered an important atherogenic protector [14,72]. A reduction in HDL-C and phospholipids could induce changes as a compensatory response which results in higher synthesis and accumulation of phospholipid-rich VLDL. In consequence, hypertriglyceridemia appears [70].

The aforementioned changes could explain some patients showing low levels of apoA-I and HDL-C after an inflammatory response [70]. Inhibition of LXR activity mediated by toll-like receptors (TLR) produces cholesterol accumulation in the vessel wall, which causes an accumulation of cholesterol-laden macrophages, causing a pro-atherogenic effect and early atherosclerotic injury [70].

Finally, alterations in function, structure, and composition of HDL-C particles may reduce CEC, antioxidant properties, and increase atherogenic effects [73].

## 5. Dietary Compounds and Their Effects on the Modulation of HDL-C Functionality

The most recent ACC/AHA 2019 Guidelines for the primary prevention of CVD recommend a healthy diet, which is characterized by high consumption of vegetables, fruits, legumes, nuts, whole grains, and fish; replacement of saturated fatty acids (SFA) with polyunsaturated fatty acids (PUFAs) and monounsaturated fatty acids (MUFAs); a reduced intake of sodium to <2400 mg/day; minimizing the consumption of processed meats, refined carbohydrates, and sweet drinks; and limit the consumption of trans fats. To achieve these recommendations, the AHA/ACC advocates for plant-based dietary patterns such as the vegetarian diet (VD) and the Mediterranean diet [31]. On the other hand, the current ES/EAS Guidelines for the management of dyslipidemias specify lifestyle modifications to improve HDL-C concentration and the available evidence on the effect of functional foods on improvements in overall lipoprotein profile concentrations [32].

Despite this, there is not enough evidence of the direct impact of these nutritional recommendations on HDL-C functionality. The effect of some of these recommendations has only been assessed concerning HDL-C concentrations [16,30,74].

Virgin olive oil, quercetin, and resveratrol participate and contribute to the improvement of cholesterol efflux from macrophages by increasing the expression of *ABCG1* and *ABCA1* transporters [75,76,77,78], while curcumin only influences the ABCA-1 transporter [79]. All of these mechanisms would prevent the accumulation of cholesterol within macrophages in the arterial wall, and its subsequent relationship with the progression of atherosclerosis. Additionally, it has been shown that curcumin, ginger, legumes, nuts, and fish can contribute to reductions in plasma CETP [80,81]. This enzyme participates in the transferring of cholesteryl esters from HDL participles to triglyceride-poor proteins, and high levels of CETP are related to CVD [82]. *PON1* expression improves the anti-oxidant, anti-inflammatory, and anti-atherogenic activity of HDL-C. Therefore, functional foods could contribute to the modulation and prevention of atherosclerosis [16] (Figure 2).

In the present paper, we summarize the evidence on HDL functionality (CEC, CETP activity, and its antioxidant capacity by PON1 activity) as well as its expression and relationship with functional foods (extra virgin olive oil [27,81,83,84,85], whole grains [81], nuts [27,81,86,87], legumes [81,88], fresh fish [81,89,90], red wine [91]), fruits and vegetables [92,93,94,95], ginger [80], green tea [91,96], cocoa [91,97], and bioactive compounds as curcumin [79,80], resveratrol [78,98], and quercetin [75,76,99].

At the moment, the evidence of dietary compounds and adhesion molecules expression is focused exclusively on an inflammatory response in a broad context, and there is no connection with the importance of HDL functionality. In this sense, the inhibition of the expression of adhesion molecules is not analyzed to narrow down the presented information in this narrative review [100,101,102].

### 5.1. Cholesterol Efflux Capacity

One of the main elements related to atherosclerosis progression is the accumulation of cholesterol into macrophages in the arterial wall, contributing to the formation of foam macrophages [103]. RCT is one of the main properties of HDL-C; it is necessary to transport accumulated cholesterol to the liver for excretion [47]. A phase of the RCT considered a fundamental aspect of atheroprotection is CEC, which refers to HDL-C’s ability to extract cholesterol from peripheral membranes, macrophages, and foam cells [50,104] by different transporters such as ABCA-1, ABCG-1, or SR-B1 [47]. CEC is used as a marker to measure HDL-C functionality [13].

There is evidence in the literature of functional foods and/or bioactive substances related to CEC, such as extra virgin olive oil [27,81,83,84], fruits [92,93], nuts [87], legumes [88], fish [89,90], quercetin [75,76], green tea, cocoa [91,97], red wine [91], curcumin [79], and resveratrol [78] (Table 1).

#### 5.1.1. Extra Virgin Olive Oil

It is the main component of the MD and is characterized by its nutritional composition, composed mainly of monounsaturated fatty acids (MUFAs), the majority being oleic acid and polyphenol compounds such as oleuropein and hydroxytyrosol [105,106].

A randomized controlled trial with a subsample from the PREDIMED study (*n* = 296), the effects of two traditional Mediterranean diets (TMD) were compared to investigate the effects of extra virgin olive oil (EVOO) and nuts on different HDL functional properties. One group received a dietary intervention that was enriched with one liter of EVOO per week (TMD-EVOO; *n* = 100). The other group received an additional 30 g of nuts per day (TMD-Nuts; *n* = 100). Both these groups were compared to a low-fat control diet (*n* = 96). After a one-year intervention, CEC levels increased relative to baseline (unitless ratio; THP-1 macrophage based model) (mean 0.01 (SD 0.07; *p* = 0.018); mean 0.02 (SD 0.09; *p* = 0.013); Table 1) [27].

In another study 33 hypercholesterolemic participants were enrolled into a crossover, double-blind, controlled intervention: the Virgin Olive Oil and HDL functionality (VOHF) study. The study assessed the relationship between CEC and changes in HDL-related variables (composition, fluidity, oxidative/antioxidative status, and particle size) after participants consumed 25 mL/day of three types of virgin olive oil (VOO): VOO (control, 80 ppm), functional olive oil enriched with its own phenolic compounds (FOO1, 500 ppm), and functional olive oil enriched with its own phenolic compounds plus thyme (FOO2, 500 ppm). The study intervention was organized into three intervention periods of three weeks each and included a two-week washout period with refined olive oil. The results showed a significant increase in a range of measures after intervention compared to pre-intervention as it is shown in the following data: % CEC (4.1(SD ± 1.4); *p* = 0.042), HDL ApoA-I concentration (0.6 (SD ± 0.1); *p* = 0.014), and antioxidants in HDL: α-tocopherol (12.7 (SD ± 0.7); *p* = 0.017), β-cryptoxanthin (8.7 (SD ± 3.4); *p* < 0.001), coenzyme-Q (324.2 (SD ± 191.1); *p* = 0.005), and phenolic compounds (63.7 (25th-75th percentile 0–588.3); *p* < 0.001). These results were independent of the type of VOO (natural VOO or enriched with phenolic compounds or thyme). CEC was inversely related with small HDL particles size (r = −0.3; *p* < 0.001), but directly related to the concentration in HDL of ApoA-I (*p* = 0.004), esterified cholesterol (r = 0.2; *p* = 0.040), medium (r = 0.2; *p* = 0.012), and large (r = 0.2; *p* = 0.041) HDL particles (Figure 2) [83].

Recently, a before–after study with a subsample of 296 high cardiovascular risk older adults (50–80 years) from the PREDIMED trial aimed to determine whether the increase in the intake of cardioprotective food groups (EVOO, nuts (walnuts, almonds, pistachios, hazelnuts, and pine nuts), fruit and vegetables, legumes, whole grains, fish, and wine) for 1 year was linked to improvements in HDL functions. The results showed that after 1 year of daily consumption of a 10 g serving (one spoonful) of EVOO was independently associated with increases in the CEC of 0.7% (0.08–1.27; *p* = 0.026) adjusted for age, sex, intervention group, clinical conditions, adherence to MD, and physical activity. The consumption of 25 g of whole grains increased CEC by 0.6% (0.1–1.1; *p* = 0.017), a similar protective effect to EVOO. However, the results could have been confounded by the high intake of dietary fiber and polyphenols [81].

Other results, in the same way, were observed by Farras et al. (2017). The consumption of phenol-enriched olive oils increased HDL-antioxidant compounds on a group of hypercholesterolemic adults. Cholesterol efflux raised 1.3% on the population of the intake olive oils high in polyphenols [84].

#### 5.1.2. Resveratrol

Resveratrol is a polyphenol present in the skin of red grapes [107]. Several researchers have identified the cardioprotective effects of resveratrol [78,107]. A study in vitro explored its potential atheroprotective impact on CE in cells of the arterial wall, including human TPH-1 monocytes and macrophages, human peripheral blood mononuclear cells (PBMC), human monocyte-derived macrophages (HMDM), and human aortic endothelial cells (HAEC). Cells were incubated for 18 h under different experimental conditions including solvent control and resveratrol concentrations of 10 μM and 25 μM. The main outcomes reported that the exposure of cells to 10 μM of resveratrol increased ABCA-1 signaling in TPH-1 and HAEC vs. control (mean 168.2% (SEM ± 13.3%); mean 141.3% (SEM ± 15.4%); *p* < 0.001), respectively. The authors express similar results were obtained in TPH-1 monocytes and PBMC. ABCG-1 upregulation was found in TPH-1 (mean 169.9(SEM ± 15.1%); *p* < 0.001), contributing to promote CE to apoA-I (20 μg/mL, 4 h) in TPH-1 up to 4.6% compared to 3.8% of solvent control (*p* < 0.05). Expression of *LXRα* (mRNA) with a concentration of 10 μM of resveratrol was increased in both TPH-1 macrophages and HAEC (mean 148.9% (SEM ± 13.3%) and (mean 125.8% (SEM ± 10.3%) of control; *p*< 0.05), respectively. The incubation of cells with 25 μM of resveratrol had an even greater effect on RCT protein expression in both cells. Just as in other studies, resveratrol regulates *PPARγ* expression versus control (mean 136.2% (SEM ± 8.5%); *p* < 0.001) [78].

Despite this, some clinical trials have recently published the effects of resveratrol (variable doses) on different chronic conditions with high cardiovascular risk. None of them was aimed to explore the role of resveratrol on CEC, and some of these studies are mentioned in the following. In a randomized, double-blind controlled trial, 56 patients with T2DM or coronary heart disease were included. The intervention group received 500 mg/day resveratrol during four weeks, and the metabolic status was investigated; the more related outcomes in relation to HDL were the increasing levels of HDL-C (β 3.38 mg/dL; 95% CI = 1.72–5.05; *p* < 0.001) and significantly decreasing total-/HDL-C ratio (β −0.36; 95% CI = −0.59–−0.13; *p* = 0.002) [108]. The effect of resveratrol on anthropometric and biochemical parameters was assessed in subjects with BMI > 30 kg/m^2^ who were supplemented with 250 mg resveratrol/day, submitted physical activity and diet regulation for three months, and compared with a placebo group. This approach addressed to control metabolic syndrome components and found increasing levels of HDL-C (*p* = 0.026) compared to placebo [109]. The long-term effect of resveratrol has also been assessed in a randomized, double-blind, placebo-controlled trial. This study was oriented to determine the impact of high and low resveratrol treatments (1000 and 150 mg resveratrol/day) on inflammation and metabolic syndrome. Unexpectedly resveratrol treatments did not improve the inflammatory status, nor did it change the HDL-C levels. High resveratrol doses increased total cholesterol (*p* < 0.002) and LDL levels (*p* > 0.006) compared to placebo [110].

#### 5.1.3. Nuts

Nuts are rich in unsaturated fat (oleic acid), polyunsaturated fatty acids (linoleic acid and α-linolenic acid), protein, dietary fiber, vitamins, and other bioactive compounds such as polyphenols [111].

Currently, other clinical trials have been performed to evaluate the intake of nuts and CEC. The objective was to examine the effect of replacing saturated fatty acids with unsaturated fats from walnuts or vegetable oils on lipoprotein subclasses, cholesterol efflux, and other markers. A randomized, crossover, controlled-feeding study was conducted with men and women (aged 30–65 years) with overweight and obesity, LDL-C between 121–177 mg/dL and/or elevated blood pressure (systolic: 120–159 mmHg; diastolic: 80–99 mm Hg), and free of chronic disease with no history of CVD. The authors compared the study variables on isocaloric diets: (1) rich in α-linolenic acid from non-walnut sources; (2) higher in monounsaturated fatty acid from oleic acid. There were no differences between diets for HDL-C or LDL-C subclasses. Cholesterol efflux capacity was unchanged after the diets [87].

#### 5.1.4. Legumes and Fish

Legumes are a great source of vegetable protein, vitamins, minerals, fibers, antioxidants, and other bioactive compounds such as phytohemagglutinin (lectins), phytoestrogens, oligosaccharides, saponins, and phenolic compounds [112]. The main phenolic compounds of legumes are phenolic acids, flavonols, flavones, isoflavones, anthocyanins, and condensed tannins [113]. On the other hand, fish oil is characterized by its high omega 3 polyunsaturated fatty acids including eicosapentaenoic acid (EPA) and docosahexaenoic acid (DHA) content [107].

In the case of fish, two trials were designed to assess its consumption effect on the atherogenic and anti-atherogenic functions of LDL and HDL particles [89] and the response to saury oil over plasma lipids [90]. Both approaches considered the CEC as the major requisite for its inclusion as exemplars in Table 1. Seventy-nine subjects with impaired glucose metabolism (40–75 years) were recruited to participate for 12 weeks in a randomized-controlled trial. Subjects were randomly assigned to *Camelina sativa* oil (CSO), lean fish, fatty fish, or control group. Each group received an isocaloric diet. Fish diets included four meals per week (an estimate consumption of 1 g eicosapentanoic acid + docasahexaenoic acid), while the CSO group ingested 30 mL of CSO (an estimate of 10 g alpha-linolenic acid). CSO and control groups were allowed to eat fish once a week. HDL particles were isolated from the subjects before and after the 12 week intervention with fatty fish to determine the CEC, and no significant modification was observed [89]. In a double-blind, randomized crossover trial, 30 healthy normolipidemic subjects (>18 years) were enrolled in an 8 week intervention study followed by 8 week washout lapse. Subjects were randomized to saury oil (12 g = 3.5 g long-chain monounsaturated fatty acid + 3.4 g omega-3 fatty acids) or control oil group (12 capsules/day with sardine + olive oil = 4.9 g monounsaturated fatty acid + 3 g omega-3 fatty acids). Both treatments had similar effects with respect to lowering plasma TG and VLDL levels (16% and 25%, respectively). It was an approximate 6% increment in HDL-C levels with both oils together with a 6% and 8% CEC rise, respectively (compared to baseline) [90].

One clinical trial was conducted to analyze the effect of a protein isolate (isoflavone-containing soya) on HDL function, macrovascular function, and blood markers. This study enrolled 20 adults (35–60 years) with moderately elevated blood pressure (129/82.5 mmHg, mean values). The subjects consumed 0, 25, and 50 g soy protein/day for 6 weeks with a 2 week washout period between treatments (three-period crossover). The soya protein isolate was obtained from soybean flakes with a content of 1.7 mg of isoflavones per g of protein, which were formulated as protein powders. The participants incorporated the powders in their diet with no changes in dietary intake and physical activity. The 50 g/d soya treatment reduced the systolic blood pressure compared to 25 g/d (−2.3 mmHg, *p* = 0.03) but not so for the control group. There were no changes in CEC, macrovascular function, or CVD markers. The authors point out the need for longer treatments and the assessment of equal production (soy metabolite) [88].

#### 5.1.5. Fruits

A recently published Word Health Organization report recommends a minimum of 400 g of fruit and vegetables per day (excluding potatoes and other starchy tubers) for the prevention of cardiovascular diseases. The evidence about intake of fruit and HDL functions is developing.

Agraz is a fruit rich in polyphenols (mainly anthocyanins). A double-blind crossover study with 40 women (25–60 years) with metabolic syndrome was carried out to evaluate the effect of agraz consumption as compared to placebo on HDL function and inflammation. Women consumed agraz or placebo over 4 weeks with a period washout at the same duration. The main results observed were that, compared to placebo, agraz consumption did not significantly change any of biomarkers including HDL functionality [93].

Another fruit with high polyphenols is grapes. The authors evaluated the effects of grape powder ingestion on measures of HDL function in 20 adults (32–70 years, BMI 25.3–45.4 kg/m^2^) with metabolic syndrome. The consumption of either 60 g/d of freeze-dried grape powder or placebo for 4 weeks, separated by a 3 week washout period, was assessed in a randomized, double-blind crossover study. The intervention group did not show significant effects on cholesterol efflux capacity and other measures [92].

#### 5.1.6. Green Tea, Cocoa, and Red Wine

They are considered foods rich in polyphenols [91] and have received special attention in cardiovascular risk prevention [91,114]. Green tea is characterized by its high levels of catechins, including epigallocatechin-gallate (EGCG), epigallocatechin (EGC), epicatechin-gallate (ECG), and epicatechin (EC) [114]. Similarly, the most abundant polyphenols in cocoa include catechins, anthocyanins, and proanthocyanins [115]. Red wine contains high concentrations of polyphenolic compounds such as resveratrol and flavonoids (catechin, epicatechin, quercetin, anthocyanin, and procyanidins) [116].

There is only one study that has investigated the association between polyphenols from green tea, cocoa, red wine, and CEC. This in vitro study used human colon carcinoma cell line (Caco-2) supplemented with a concentration of 50 μM of total polyphenols (red wine, green tea, or cocoa extracts) calculated as gallic acid equivalents. The main aims were to assess the effect of polyphenols on intestinal inflammation, using Caco-2 monolayer model, and to investigate the action mechanisms of the increase in HDL-C by polyphenols. This study did not find any effect on cholesterol secretion or cholesterol uptake by SR-B1 or HDL functionality after 24 h with 50 μM of total polyphenols. However, the study suggests that polyphenols from red wine and green tea could modulate intestinal inflammation by the reduction in basolateral IL-6 secretion by Caco-2 cells grown on Transwells and LPS-treated (*p* < 0.05). Nevertheless, the authors concluded that the effects were moderate, and there was variability in the results of different experiments. Finally, red wine, cocoa, and green tea administered at a dietary dose did not increase cholesterol secretion by intestinal cells nor enhanced HDL functionality [91].

#### 5.1.7. Curcumin

Curcumin is a bioactive polyphenol, a yellow pigment of *Curcuma longa* (turmeric), often used as a spice in India and traditional Chinese medicine [79,117,118]. It has been considered to have antioxidant, anti-inflammatory, anti-angiogenic, and anticancer properties [117,118].

Few research studies have investigated the effects of curcumin on improving HDL functionality regardless of HDL-C concentration [117]. An in vitro study in RAW264.7 and TPH-1 macrophages explored the effect of different concentrations of curcumin (10, 20, 40 μM) on CEC. The authors concluded that curcumin had a dose-dependent effect on cholesterol efflux, and the %CEC was approximately duplicated in both cell lines (*p* < 0.05) compared to the control group. It was additionally observed that RAW264.7 and TPH-1-derived macrophages treated with all the different concentrations of curcumin for 12 h increased *ABCA1* and *SRB1* expression and protein level vs. data in the control group (*p* < 0.05), which promoted cholesterol efflux [79].

#### 5.1.8. Quercetin

Quercetin is part of a group that comprises over 4000 naturally available plant phenolic compounds and is considered a flavonol, a type of flavonoid [75,99]. Its main food sources are vegetables and fruits such as elderberries, red onions, white onions, cranberries, green hot pepper, and red apples, among other foods [99,119].

Molecular studies in cell culture treated with quercetin demonstrated that quercetin increased CE from macrophages through the expression of *ABCA1* in a dose and time-dependent manner [75]. An in vitro study used treated foam cells derived from oxLDL-induced TPH-1 cells with different doses of quercetin (0, 25, 50, 100, 200 µM) and periods (0, 4, 8, 16, 24, 32 h) to investigate the function of quercetin on CE from foam cells, *ABCA1* and *PPARγ* expression. The results indicated that higher doses and longer exposure to quercetin resulted in a greater increase in apoA-I-dependent CEC ((200 µM, ~20% increase in CEC); (32 h, >20% in CEC)) and induced *ABCA1* expression at mRNA and protein levels in THP-1-derived foam cells (200 µM, *p* < 0.01; *p* < 0.001), respectively. Quercetin increased the expression and activation of *PPARγ* (PPARγ mRNA and protein levels; 200 uM, 32 h, *p* < 0.001) by ~3 times in a dose- and time-dependent manner. PPARγ upregulates *ABCA1* expression [75].

In the same way, an in vivo study in a model of 24 apoE-deficient mice fed with a high-fat diet was divided into two groups: carboxymethyl cellulose sodium by gavage (CMCNa) group (*n* = 12, 0.5% CMCNa) and the quercetin group (12.5 mg/kg/d in 0.5% CMCNa by gavage) to investigate whether quercetin improves RCT in an atherosclerosis model of “apoE-/- mice” and the underlying mechanism by molecular techniques. The outcomes showed that CEC from macrophage to plasma increased 31.8% and HDL 22.1% in the quercetin-treated mice compared with the controls (*p* < 0.01). The authors concluded that quercetin improved CEC by upregulating the protein expression of the main transporters in RCT, ABCA-1, and ABCG-1 at a concentration of 2.5 μM, and also by elevating the cholesterol-accepting ability of HDL and apoA-I via reduction in oxidation (Figure 2) [76].

### 5.2. Activity of Cholesteryl Ester Transfer Protein (CETP)

CETP is an enzyme that plays a major role in lipid metabolism. It participates in transferring cholesteryl esters (CE) from HDL particles to triglyceride-poor lipoproteins (and subsequently to the liver) [82]. CETP activity has been associated with some dietary compounds (Table 2) such as legumes, fresh fish [81], EVOO [27,81], curcumin, and ginger [80].

#### 5.2.1. Extra Virgin Olive Oil

The subsample from the PREDIMED clinical trial in 296 older adults compared three intervention groups to investigate its effects on improving HDL functionality: supplementation with one liter of EVOO per week (TMD-EVOO), 30 g of nuts per day (TMD-Nuts), and a control group with a low-fat diet. This research demonstrated that CETP activity decreased significantly after the TMD-EVOO intervention compared to baseline (−0.039; *p* = 0.008) [27].

#### 5.2.2. Legumes and Fresh Fish

In a study subsample of 296 high cardiovascular risk older adults from the PREDIMED trial, researchers aimed to determine if the increases in the intake of EVOO, fruit, vegetables, legumes, whole grains, fish, and wine for 1 year were linked to improvements in HDL functionality including CETP activity. The study found an increase in legume intake (25 g per day, 2 servings per week) was associated with a 4.8% decrease in CETP activity (*p* = 0.0028), and an increase in fish consumption (25 g/d, 2 servings per week) was linked to a -1.6% decline in CETP activity (*p* = 0.021) after 1 year of intervention. Compared with fish subtypes, only fresh fatty fish consumption was related to a greater decrease in CETP activity (−2.3%; *p* = 0.043). No significant results were found in HDL functionality with the fruits, vegetables, and wine intake [81].

#### 5.2.3. Curcumin and Ginger

Curcumin and ginger are characterized by their anti-atherogenic properties. Elseweidy et al. [80] in their experimental animal model study used hypercholesterolemic rabbits. They compared the atheroprotective potentials of total ginger extract (TGE), or curcuminoids extracted from turmeric, on lipoprotein profile, CETP, hepatic cholesterol, inflammatory, and oxidation biomarkers. Rabbits (*n* = 6) fed on a high-cholesterol diet for 6 weeks received three types of intervention (TGE, curcuminoids, and placebo). It resulted in lower hepatic *CETP* mRNA expression (8.4(SD ± 0.5), 8.7(SD ± 0.7), 11(SD ± 0.5), respectively; *p* < 0.001) and plasma CETP (152(SD ± 5) pg/mL, 199 (SD ± 4), vs. 315(SD ± 12), respectively; *p* < 0.001) compared with placebo. Ginger was more effective than curcuminoids at decreasing plasma CETP (152 pg/mL (SD ± 5) vs. 199 (SD ± 4); *p* < 0.001), but not at its gene expression (Table 2). Curcuminoids and TGE decreased hepatic cholesterol (mean 28 mg/g (SD ± 0.7); 21 (SD ± 1) vs. 30 (SD ± 0.1); respectively), LDL-C (mean 184 mg/dL (SD ± 13); 119 (SD ± 8) vs. 373(SD ± 52)) and increased HDL-C (mean 36.8 mg/dL (SD ± 3.4); 32.2 (SD ± 4.8) vs. 17.6 (SD ± 0.7); *p* < 0.001) compared with placebo. The authors concluded that the effects of curcuminoids and ginger are mediated by different mechanisms. TGE presents better results on plasma lipids, RCT, cholesterol synthesis, and inflammatory status; curcuminoids showed better antioxidant activity [80].

### 5.3. Antioxidant Capacity: PON1 Activity/Expression

The PON is a group of three members (PON1-3) of antioxidant enzymes associated with apoA-I contained in HDL. PON1 and PON3 are associated with cardiovascular health benefits by promoting cholesterol efflux to HDL and preventing lipoprotein oxidation [85].

The consumption of olive oils [27,81,85], nuts, fruit and vegetables [92,93,94,95], legumes, and fish [81] are associated with PON1 activity or expression (Table 3).

#### 5.3.1. Olive Oil

Two randomized, crossover-controlled trial interventions and an experimental design with rats were used by Fernández-Castillejo and colleagues [85]. The studies evaluated the short- and long-term modulation of acute and sustained intake of olive oils according to their PC content and source on PON-related variables and the mechanism involved in the OO-PC and thyme-PC effects on such variables. The mechanisms related to PON1 modulation were assessed with an experimental procedure.

In the first acute intake study, twelve healthy subjects were divided into three interventions with a single dose of 30 mL of functional virgin olive oil (FVOO) with different phenolic compounds (PC) content: low-FVOO (L-FVOO, 250 ppm), medium-FVOO (M-FVOO, 500 ppm), high-FVOO (H-FVOO, 750 ppm). Blood samples were collected at baseline and 2, 4, and 6 h postprandial. It revealed that 30 mL of L-FVOO and M-FVOO decreased PON1 protein levels after 2 h of OO intake (5.1–6.4%; *p* < 0.05), whereas H-FVOO promoted an increase in PON1 (6.8%) at this time point vs. baseline without statistical significance. L-FVOO and M-FVOO intake increased paraoxonase raw activity at the 2 h time point followed by a decrease at 4 h time point (*p* < 0.05) [85].

The second study, a sustained intake study, was an intervention in 33 hypercholesterolemic volunteers, for three weeks with an intake of 25 mL per day of a control VOO and two different FVOO with the same PC content but differing in PC source: FVOO (80 ppm)+ olive oil oleuropein derivatives as hydroxytyrosol (OO-PC) as a control, VOO enriched with its own PCs (FVOO) (500 ppm, 50% secoiridoid derivatives), and VOO enriched with its own PCs plus thyme (FVOOT) (50% flavonoids, phenolic acids, and monoterpenes). It found that PC intake led to a decrease in PON1 protein levels by 10.9 and 12.4% after VOO and FVOO respectively, while it also increased the PON3 protein levels by 5.1% (*p* < 0.05) and PON1 catalytic activity (*p* < 0.05). The mixture of OO-PC and FVOOT with thyme produced the opposite results [85].

The experimental design was a procedure with 20 male and female Wistar rats randomized into four groups: control diet (CD), CD supplemented with OO-PC extract (secoiridoids or hydroxytyrosol derivatives), diet supplemented with thyme phenol content extract (THY), and diet supplemented with secoiridoids and thyme extracts (SEC + THY). The rats ingested 5 mg of phenolic extract/kg/d for 21 days. The results of the animal model showed that *Pon1* gene expression was correlated with PPARγ (r = 0.966; *p* = 0.034) and increased *Pon3* hepatic gene expression in SEC group vs. CD group, while THY intake decreased *Pon1* and *Pon3* vs. SEC group. The authors suggested these changes in PON enzymes may reflect an improvement in HDL functionality as indicative of adequate oxidative balance [85].

In another crossover, double-blind, controlled trial which analyzed 33 hypercholesterolemic participants from VOHF after 3 weeks of intervention with different types of VOO, the authors did not find a significant change in PON1 activity relative to baseline after one year of intervention with VOO (1 L/week) [83]. These results were confirmed in another study using a subsample of the PREDIMED trial where there was no significant change in PON1 arylesterase activity, after 1 year of intervention with TMD enriched with olive oil (1 L/week) [27].

#### 5.3.2. Fruits, Vegetables, and Resveratrol

Four randomized controlled studies, three with a control group, were carried out to assess the intake of fruit (2 juice [94,95], 2 fruit freeze-dried [92,93]) on PON1 activity. The range time of the intervention was 4–12 weeks. The participants were subjects with hypertension, diabetes, or metabolic syndrome (20–40 years). Three studies reported no significant changes between the intervention vs. the control group after the nutritional intervention, or at the end of the period intervention. Only Lazavi et al., 2018, showed that after 8 weeks of intake of barberry juice (200 mL) a significant increase in PON1 concentration (56 mg/dL; *p* = 0.015) was seen compared with the control group [95].

The consumption of green tea or yerba mate has been evaluated on functions of HDL, in this case PON1 activity. A randomized clinical trial on 142 subjects with overweight or obesity and dyslipidemia determined PON1 activity on three intervention groups: intake 1000 mL/d of green tea, 1000 mL/d of yerba mate, or 1000 mL/d apple tea. After 8 weeks of intervention, the authors observed a significant increase of 19.7% (*p* < 0.05) PON1 serum concentration in the yerba mate group compared with the other groups. Green tea intervention group results were not changed [96].

One clinical trial with supplementation of resveratrol was found. Seventy-one patients with type 2 diabetes were randomized to intake 2000 mg of resveratrol or placebo for 8 weeks, and PON1 activity was evaluated in blood. After this period of intervention, PON1 increased its activity significantly compared to the placebo group [98]. Data are not shown in the tables because the study did not evaluate the food intake.

#### 5.3.3. Nuts, Fish, and Legumes

Nuts are rich in unsaturated fats (oleic acid), polyunsaturated fatty acids (linoleic acid and α-linolenic acid), protein, dietary fiber, vitamins, and other bioactive compounds such as polyphenols [111].

A subsample of 296 high cardiovascular risk older adults from the PREDIMED clinical trial evaluated the intake of EVOO, nuts (walnuts, almonds, pistachios, hazelnuts, and pine nuts), fruit and vegetables, legumes, whole grains, fish, and wine on PON1 antioxidant activity. After 1 year, authors found improvements in PON1 antioxidant activity by 12.2% (*p* = 0.049), 11.7% (*p* = 0.043), and 3.9% (*p* = 0.030) after the consumption of 30 g of nuts per day (a small fistful), 25 g legumes per day (2 servings per week), and 25 g per day (2 servings per week) of fish, respectively [81].

## 6. Conclusions

The worldwide prevalence of low HDL-C among adults over 19 years is around 25%, and it is a risk factor considered an important indicator to develop atherosclerosis. The number of people affected by dysfunctional HDL-C is still seldom studied.

There is not enough evidence at present about the association of HDL-C functionality and dietary compounds, and there are even fewer data regarding the suggested therapeutic doses of bioactive compounds to protect against cardiovascular diseases. More human studies are needed to investigate the effect of functional foods and their bioactive compounds on HDL alterations to develop nutritional precision recommendations for daily allowances to incorporate into the diet. Such evidence may be useful as part of a wider suite of prevention and management guidelines to reduce the global burden of cardiovascular disease.

In our review, we presented the studies available in the literature concerning three mechanisms of action of HDL-C, the anti-inflammatory HDL functions, and the evidence is even more limited. In summary, we found the following results for cholesterol efflux capacity: 3 randomized controlled trials, 3 randomized crossover-controlled trial, 2 randomized double-blind crossover-controlled trials, 1 randomized double-blind crossover trial, 1 crossover, double-blind, controlled trial, 1 double-blind crossover study, 4 in vitro, and 1 in vivo studies. The activity of cholesteryl ester transfer protein (CETP) included 2 randomized controlled trials and one in vivo study. Antioxidant capacity involving PON1 activity/expression included 3 randomized controlled trials, 1 randomized double-blind controlled trial, 1 randomized single-blind study, 1 randomized clinical trail, 1 randomized crossover-controlled trial, 1 randomized double-blind crossover-controlled trial, and 1 double-blind crossover trial. The critical examination of the trials published to date illustrates that intake of functional food and bioactive compounds leads to moderate or relevant improvements in HDL functions in high cardiovascular risk subjects. It is important to note that, in most of the analyzed clinical trials, the main objective was not to study HDL functionality, and the results were derived from sub-studies.

Future scientific research has to strengthen the knowledge of the mechanisms between HDL-C and dietary compounds, as well as to the efficacy of functional foods and bioactive components in preventing or curtailing HDL dyslipidemia. It will involve the achievement of all types of basic and applied study designs, such as in vivo, in vitro, observational and clinical trials. Well-defined designs are needed with the specific proposals to the study HDL functions and concentrations, different populations with specific selection criteria, precise dose, and well-characterized bioactive components or food along with specific dietary patterns, planned endpoints, and several extended follow-ups. New research opportunities would include analysis of concentration and expression of HDL-C subfractions, HDL-Lp-PL-A2, and adhesion molecules linked with HDL-C. With this evidence collected, we can then reach the goals to have clinical and public health indicators of HDL-C functionality to prevent CVD, beyond just concentration.

## Figures and Tables

**Table 1:** Studies regarding dietary compounds effects on cholesterol efflux capacity of HDL-C *.

Author, Year	Dietary Compounds	Dose/Time	Study Design *n*	Main Results on Efflux Capacity on HDL-C
Hernáez, 2017 [27]	TMD enriched with EVOO TMD enriched with nuts (walnuts, hazelnuts, and almonds)	1 L/week 30 g/d (15 g walnuts, 7.5 g hazelnuts, 7.5 g almonds) 1 year	A randomized controlled trial subsample PREDIMED study 296 subjects (TMD-EVOO; *n* = 100 and TMD-Nuts; *n* = 100, low-fat control diet; *n* = 96).	↑ CEC TMD-EVOO interventions relative to baseline (0.01 ± 0.007; *p* = 0.018) ↑ CEC TMD-Nuts interventions relative to baseline (0.02 ± 0.09; *p* = 0.013)
Hernáez, 2019 [81]	EVOO Whole grains	10 g/d (one spoonful) 25 g/d 1 year	A randomized controlled trial subsample PREDIMED study 296 older adults’ high cardiovascular risk (50–80 years)	↑ 0.7 % CEC (0.08–1.2; *p* = 0.026) with EVOO ↑0.6% CEC (0.1–1.1; *p* = 0.017) with whole grains.
Fernández-Castillejo, 2017 [83]	VOO (80 ppm) FVOO enriched with its own PC (500 ppm) FVOOT with its own PC plus thyme (500 ppm)	25 mL/day 3 weeks	Crossover, double-blind, controlled trial from the VOHF 33 hypercholesterolemic subjects	↑ CEC post-intervention vs. pre-intervention values (4.1% ± 1.4; *p* = 0.042). ↑ HDL ApoA-I concentration (0.6 ± 0.1; *p* = 0.014). Independent of VOO type. CEC was related to concentration in HDL of ApoA-I (*p* = 0.004).
Farràs, 2017 [84]	VOO (80 ppm) FVOO enriched with its own PC (500 ppm) FVOOT with its own PC plus thyme (500 ppm)	25 mL/day raw OO (between meals) 3 weeks 2 weeks wash-out periods	Randomized, double-blind, crossover, controlled trial from the VOHF 33 hypercholesterolemic subjects	FVOOT versus FVOO intervention ↑ CEC (1.3% ± 3.9 and 1.2% ± 3.8, respectively; *p* = 0.019) FVOOT versus VOO ↓ (−0.03% + 5.4) FVOOT post-intervention versus baseline. ↑ CEC (29.7% ± 5.6 vs. 28.3% ± 6.7; *p* = 0.086)
Tindall, 2019 [87]	Walnuts Vegetable oils	WD WFMD ORAD	Randomized, crossover, controlled-feeding study 34 individuals at risk of cardiovascular disease (aged 30–65 years)	~ CEC mediated for ABCA1 (*p* = 0.1) or global efflux in all diets (WD 3.5% ± 0.2, WFMD 3.5% ± 0.2, ORAD 3.8% ± 0.2; *p* = 0.1). ↓ global efflux after the WFMD compared with WD and ORAD (*p* = 0.01).
Manninen, 2019 [89]	Fish Camelina sativa oil	20 mL of CSO * 4 meals/week of lean Fish 1 g EPA + DHA per day of fatty fish Control * * CSO and control allow one fish per week 12 weeks	Randomized controlled trial 79 impaired glucose metabolism subjects (40–75 years)	~ CEC of HDL (*p* = 0.123) had no significant effect after 12 weeks of fatty fish ingestion.
Yang, 2019 [90]	Fish LCMUFA, omega-3 FA, MUFA	12 g saury oil, control oil (sardine + olive oil)	Randomized, doble blind, crossover trial 30 healthy normolipidemic subjects [>18 years, (34.8 ± 12.5)] 8 weeks	↑ 6.2% HDL-C levels, ↑ 8% CEC
Richter, 2017 [88]	Soya protein (isoflavone)	0, 25 and 50 g/day soya protein 8 weeks	Randomized, placebo-controlled, three period crossover study 20 adults with moderately elevated blood pressure (35–60 years)	~ CEC No significant effects in CE ex vivo. ↓ 12.7 % ABCA1-specific efflux (*p* = 0.02) from baseline following supplementation with the control Change not significant compared with ABCA1 efflux by 50 g/day of soya protein (3.1%; *p* = 0.4).
Millar, 2018 [92]	Grape	60 g/day of freeze-dried grape powder (GRAPE, 195 mg polyphenols) 60 g/day of placebo powder (without polyphenols) 4 weeks 3 weeks washout	Randomized, double-blind, crossover placebo-controlled study 20 adults with MS (aged 32–70 years)	~ CEC after interventions with grape and placebo (15.1% ± 5.0 and 14.4 ± 5.5; respectively). Grape not affect HDL CEC compared with placebo (0.7 ± 4.2; *p* = 0.47)
Marín-Echeverri, 2018 [93]	Agraz (fruit)	200 mL freeze-dried agraz reconstituted/day Placebo (similar beverage without any polyphenols) 12 weeks	Double-blind crossover study 40 women with MS	~ CEC (0.5% ± 2.9; *p* = 0.324) after comparing the end of both intervention periods (placebo versus agraz)
Talbot, 2018 [97]	Cocoa (theobromine)	20 mL drink (500 mg of theobromine) 20 mL placebo drink per day 4 weeks	Randomized, double-blind, controlled, crossover study 44 overweight and obese subjects (aged 45–70 years)	Not affect fasting CEC after theobromine intervention (+0.4% point; −2.81, 3.57; *p* = 0.81). ~ CEC after theobromine on fasting and postprandial CEC (97.5% ± 9.2 to 99.1 ± 11.7).
Nicod, 2014 [91]	Polyphenols (red wine, cocoa, or green tea)	50 μM total polyphenols (gallic acid equivalents) 24 h	In vitro study Caco-2 monolayer model	No change of cholesterol efflux, via SR-B1 (cholesterol is taken up by SR-B1)
Voloshyna, 2013 [78]	Resveratrol	10, 25 μM 4 h (CEC of ApoA-1) 6 h (CEC to HDL)	In vitro study TPH-1 monocytes and macrophages, HAEC, PBMC, HMDM 18 h	↑ *ABCA1* message (10 μM) in TPH1 and HAEC vs. control (168.2 ± 13.3; 141.3 ± 15.4%; *p* < 0.001) ↑ *ABCG1* expression in TPH-1 (169.9 ± 15.1%; *p* < 0.001) ↑ *LXRα* mRNA (10 μM) in TPH-1 and HAEC vs. control (148.9 ± 13.3% vs. 125.8 ± 10.3%; *p* < 0.05) ↑ 4.6% CEC to ApoA-1 in TPH-1 (20 μM/mL, 4 h) vs. 3.8% control (*p* < 0.05). ↑ 136.2 (±8.5%; *p* < 0.001) *PPARγ* expression vs. control
Sun, 2015 [75]	Quercetin	0, 25,50, 100, 200 μM 0, 4, 8, 16, 24, 32 h	In vitro study TPH-1 derived foam cells	200 uM, 32 h ↑ ApoA-I dependent CEC after 200 μM, 32 h vs. without treatment (>30% vs. 10%; *p* < 0.001) ↑ *PPARγ* expression and activation (*p* < 0.001) in 200 μM, 32 h.
Cui, 2017 [76]	Quercetin	Quercetin 12.5 mg/Kg/d in 0.5% CMCNa 2.5, 5.0, 10.0 μM 8 weeks	In vivo study Experimental animal model (apoE-deficient mice fed a high-fat diet) 24 mice CMCNa group (*n* = 12), quercetin group (*n* = 12).	↑ 31.8% CEC from macrophages in the quercetin-treated mice vs. controls (*p* < 0.01) ↑22% HDL in quercetin group (*p* < 0.01) ↑ CEC in a concentration-depend manner 5.0 and 10.0 μM (*p* < 0.01)
Zhong, 2017 [79]	Curcumin	10, 20, 40 μM 12 h	In vitro study Murine macrophage RAW264.7 cell line and monocyte TPH-1 cell line	↑ CEC in macrophage in a dose-dependent manner (10, 20, 40 μM) vs. untreated group (*p* < 0.05). ↑ *ABCA1* and *SRB1* expression and protein level (10, 20, 40 μM) vs. control group (*p* < 0.05). ~ *SRB1* expression.

Abbreviations and symbols: TMD, traditional Mediterranean diet; EVOO, extra virgin olive oil; PREDIMED, PREvención con DIeta MEDiterránea; TMD-EVOO, traditional Mediterranean diet enriched with extra virgin olive oil; TMD-Nuts, traditional Mediterranean diet enriched with nuts; CEC, cholesterol efflux capacity; TPH-1, human acute monocyte leukemia cells line; VOO, virgin olive oil; FVOO, functional virgin olive oil; PC, phenolic compounds; FVOOT, functional virgin olive oil plus thyme; VOHF, virgin olive oil and HDL functionality; Fu5 AH, macrophages and rat hepatoma cell; HMDM, human monocyte-derived macrophages; ABCA-1, ATP binding cassette subfamily A member 1; ABCG-1, ATP binding cassette subfamily G member 1; WD, walnut diet; WFMD, walnut fatty acid-matched diet; ORAD, oleic acid replaces ALA diet; CSO, camelia sativa oil; EPA, eicosapentanoic acid; DHA, docosahexaenoic acid; HDL, high-density lipoprotein; MS, metabolic syndrome; ApoA-I, apolipoprotein A-I; HAEC, human aortic endothelial cells; PBMC, human peripheral blood mononuclear cells; LXRα, liver X receptor alfa; PPARγ, peroxisome proliferator-activated receptor; Caco-2; human colon carcinoma cell line; SRB1, scavenger receptor class B type 1; CMCNa, carboxymethyl cellulose sodium; LCMUFA, long-chain monounsaturated fatty acid; MUFA, monounsaturated fatty acid; FA, fatty acid; ALA; alpha-linolenic acid. ~ not significant differences. * In vitro studies were performed independent of the study design for the CEC assessment (different cell lines included). ↓: decreasing; ↑: increasing.

**Table 2:** Studies on dietary compounds effects on the activity of CETP of HDL-C.

Author, Year	Bioactive Compounds	Dose/Time	Study Design *n*	Main Results on CETP Activity
Hernáez, 2017 [27]	TMD- EVOO TMD- Nuts	1 L/week 1 year	Randomized controlled trial subsample PREDIMED study 296 subjects (TMD-VOO; *n* = 100 and TMD-Nuts; *n* = 100, low-fat control diet; *n* = 96).	↓ CETP activity after TMD-EVOO intervention to baseline (−0.039; *p* = 0.008).
Hernáez, 2019 [81]	Legumes Fresh fish	25 g/d (2 servings/week) each one 1 year	Randomized controlled trial subsample PREDIMED study 296 older adults of high cardiovascular risk (50–80 years)	25 g legumes ↓ 4.8% (*p* = 0.0028) CETP activity 25 g fish consumption ↓ 2.3% CEPT activity
Elseweidy, 2015 [80]	Curcuminoids and ginger	50 mg/kg/d 200 mg/kg/d 6 weeks	In vivo Study Experimental animal model (rabbit model) Fed high-cholesterol diet 6 weeks 3 groups: 1.TGE (*n* = 6) 2. Curcuminoids (*n* = 6) 3. Placebo (*n* = 6)	↓ hepatic *CETP* mRNA expression TGE, curcuminoids vs. placebo (8.7 ± 0.7; 8.4 ± 0.5 vs. 11 ± 0.5; *p* < 0.001); respectively. ↓ plasma CETP (199 pg/mL ± 4; 152 ± 5 vs. 315 ± 12; *p* < 0.001); respectively. Ginger was more effective in ↓ plasma CETP (152 pg/mL ± 5 vs. 199 ± 4; *p* < 0.001) than curcuminoids.

Abbreviations and symbols: HPCOO, high polyphenol content olive oil; LPCOO, low polyphenol content olive oil; CETP, cholesterol ester transporter protein; SD, standard deviation; TMD-EVOO, traditional Mediterranean diet enriched with extra virgin olive oil; TMD-Nuts, traditional Mediterranean diet enriched with nuts; PREDIMED, PREvención con DIeta MEDiterránea; TGE, total ginger extract; mRNA, messenger RNA. ↓: decreasing.

**Table 3:** Studies on dietary compounds effects on antioxidant capacity of HDL-C through PON1.

Author, Year	Bioactive Compounds	Dose/Time	Study Design *n*	Main Results on PON1 Activity/ Expression
Michaličková, 2019 [94]	Polyphenol-enriched tomato Juice	IG: 200 g tomato fruit juice enriched with 1 g of ethanolic extract or whole tomato fruit CG: 200 g tomato fruit juice 4 weeks	Randomized controlled single-blind study 26 subjects (aged 45–60 years) with Stage 1 Hypertension	~ PON1 in both groups No significative changes baseline and 4 weeks after IG and CG [157 U/L (141–541)-172 U/L (157–447); 413 U/L (264–484)-405 U/L (294–514)]; *p* = 0.769
Lazavi, 2018 [95]	Barberry juice	IG: 200 mL/d of BJ CG: no intervention 8 weeks	Randomized clinical trial 41 diabetic subjects (aged 30–75 years)	↑56.0 mg/dL PON1 concentrations (±68.29; *p* = 0.015) for IG at the end of the trial.
Millar, 2018 [92]	Grape	60 g/d of freeze-dried grape powder (GRAPE, 195 mg polyphenols) 60 g/d of placebo powder (without polyphenols) 4 weeks 3 weeks washout	Randomized, double-blind, crossover placebo-controlled study 20 adults with MS (aged 32–70 years)	~ PON1 arylesterase and PON1 lactonase activities after interventions with grape and placebo (84.5 kU/L ± 17.4 and 86.3 kU/L ± 16.2) and (15.8 kU/L ± 3.2 and 15.6 kU/L ± 2.5; respectively) Grape not affect PON1 lactonase activity compared with placebo (0.2 kU/L ± 1.8; *p* = 0.6).
Tabatabaie, 2020 [98]	Resveratrol	2 capsules (1000 mg) of resveratrol per day 2 capsules of methylcellulose (placebo) per day 8 weeks	Randomized, double-blind controlled trial 71 patients with type 2 diabetes (aged 30–60 years)	↑ PON1 activity after supplementation with resveratrol (15.3 U/L ± 13.9; *p* < 0.001) and compared with placebo group (*p* = 0.04) Significantly after adjusting confounding variables (*p* < 0.001).
Marín-Echeverri, 2018 [93]	Agraz (fruit)	200 mL freeze-dried agraz reconstituted/day Placebo (similar beverage without any polyphenols) 12 weeks	Double-blind crossover study 40 women with MS (aged 25–60 years).	~ PON1 arylesterase and lactonase activities (-0.7 kU/L ± 8.8, *p* = 0.643; 0.2 kU/L ± 1.6, 0.862) after comparing the end of both intervention periods (placebo versus agraz); respectively.
Hernáez, 2017 [27]	TMD- EVOO TMD- Nuts	1 L/week 1 year	Randomized controlled trial subsample PREDIMED Study 296 subjects (older adults) TMD-EVOO TMD-Nuts Low-fat control diet.	~ PON1 in both groups
Hernáez, 2019 [81]	EVOO Nuts Legumes Fish	1 L/week 30 g per day 25 g per day 25 g per day 2 servings/week each (one) 1 year	Randomized controlled trial PREDIMED Study 296 older adult high cardiovascular risk (aged 50–80 years)	Nuts, legumes and fish ↑ 12.2%, 11.7% and 3.9% PON1 antioxidant activity (0.13–24.2; *p* < 0.049; 0.44–22.8; *p*= 0.043; 0.40–7.45; *p* = 0.030); respectively.
Fernández-Castillejo, 2017 [85]	First Study (acute intake) FVOOT (different concentrations) Second Study (sustained intake) Olive oil PC Thyme PC	30 mL single dose L-FVOO 250 ppm M-FVOO 500 ppm H-FVOO, 750 ppm 25 mL per day FVOO (80 ppm) + OO-PC control FVOO (550 ppm) own PC FVOOT (550 ppm) own PC (50% secoiridoid derivatives) FVOOT plus thyme (50%; flavonoids, PC, and monoterpenes)	Two randomized, crossover-controlled trial 12 healthy subjects and 33 hypercholesterolemic subjects; respectively. Single-dose and 3 weeks	↓ PON1 protein after 2 h of 30 mL of L-FVOO and M-FVOO (5.1–6.4%; *p* < 0.005) ↑PON1 raw activity at 4 h time point (*p* < 0.05). ↓ 10.9–12.4% PON1 protein levels after VOO and FVOO (*p* < 0.05) ↑ 5.1% PON3 protein levels and PON1 catalytic activity (*p* < 0.05) *Pon1* gene expression correlated with PPARγ (r = 0.966; *p* = 0.034).
Balsan, 2019 [96]	Green tea Yerba mate	1000 mL per day of: GT YM AT (control) 8 weeks	Randomized, controlled, clinical trial 142 overweight or obesity and dyslipidemia (aged 35–60 years)	↑9.7% PON1 serum levels after YM intervention (2625 pg/mL to 2880 pg/mL, change 255 pg/mL; *p* = 0.005). ~ PON1 serum levels after green tea intervention (2899 pg/mL to 2745 pg/mL, change -154 pg/mL; *p* = 0.154).

Abbreviations and symbols: IG, intervention group; CG, control group; BJ, barberry juice; MS, metabolic syndrome; FVOOT, functional virgin olive oil; PC, phenolic compounds; L-FVOO, low-functional olive oil; M-FVOO, medium-functional olive oil; H-FVOO, high-functional olive oil; OO-PC, olive oil oleuropein derivatives; PC, phenolic compounds; PON1, paraoxonase 1; PON3, paraoxonase 3; PPARγ, peroxisome proliferator-activated receptor; TMD-EVOO, traditional Mediterranean diet enriched with extra virgin olive oil; TMD-Nuts, traditional Mediterranean diet enriched with nuts; PREDIMED, PREvención con DIeta MEDiterránea Study; DP, dietary pattern; GT, green tea; YM, yerba mate; AT, apple tea. ~ not significant differences. ↓: decreasing; ↑: increasing.

## Data Availability

No new data were created or analyzed in this study. Data sharing is not applicable to this article.

## Abbreviations

ABCA-1	ATP-binding cassette transporter A member 1
ABCG-1	ATP-binding cassette transporter G member 1
ABCG-4	ATP-binding cassette transporter G member 4
ACC/AHA	American College of Cardiology/American Heart Association
ApoA-I	Apolipoprotein A-I
Caco-2	Human colon carcinoma cell line
CE	Cholesterol efflux
CEC	Cholesterol efflux capacity
CETP	Cholesteryl ester transfer protein
CMCNa	Carboxymethyl cellulose sodium
CVD	Cardiovascular diseases
DHA	Docosahexaenoic acid
EC	Epicatechin
ECG	Epicatechin-gallate
EGC	Epigallocatechin
EGCG	Epigallocatechin-gallate
EPA	Eicosapentaenoic acid
EVOO	Extra virgin olive oil
Fu5AH	Macrophages and rat hepatoma cell
FVOO	Functional virgin olive oil
H-FVOO	High- functional olive oil
HAEC	Human aortic endothelial cells
HDL-C	High-density lipoprotein cholesterol
HMDM	Human monocyte-derived macrophage
HPCOO	High polyphenol compound olive oil
ICAM-1	Intercellular adhesion molecule-1
IDL-C	Intermediate-density lipoprotein cholesterol
IL-1	Interleukin 1
IL-8	Interleukin 8
L-FVOO	Low-functional olive oil
LCAT	Lecithin cholesterol acyltransferase
LDL-C	Low-density lipoprotein cholesterol
LPCOO	Low polyphenol compound olive oil
LPS	Lipopolysaccharides
LXR	Liver X receptor
LXR-α	Liver X receptor alfa
M-CSF	Macrophage colony-stimulating factor
M-FVOO	Medium-functional olive oil
MCP-1	Monocyte chemoattractant protein 1
MD	Mediterranean diet
MPO	Myeloperoxidase
MUFAs	Monounsaturated fatty acids
NFk- β	Factor kappa β
NO	Nitric oxide
OO-PC	Olive Oil-Phenolic compounds
ox-LDL	Oxidized LDL
PAF-AH	Platelet-activating factor-acetylhydrolase
PBMC	Human peripheral blood mononuclear cell
PC	Phenolic compounds
PON1	Paraoxonase 1
PPARs	Peroxisome proliferation-activated receptors
PREDIMED	PREvención con DIeta MEDiterránea
PUFAs	Polyunsaturated fatty acids
RCT	Reverse cholesterol transport
ROS	Reactive oxygen species
SAA	Serum amyloid A
SEC + THY	Secoiridoid and thyme extracts
SFA	Saturated fatty acids
SMC	Smooth muscle cells
sPLA2-IIA	Secretory phospholipase A2
SR-B1	Scavenger receptor class B type 1
TGE	Total ginger extract
THY	Thyme phenol content extract
TLR	Toll-like receptors
TMD	Traditional Mediterranean diet
TNF	Tumor necrosis factor
TPH-1	Human acute monocyte leukemia cells line
VCAM-1	Vascular adhesion molecule-1
VD	Vegetarian diet
VLDL-C	Very low-density lipoprotein cholesterol
VOHF	Virgin olive oil and HDL functionality
